# A modification of the Hilger Analytical Chemispek multichannel electrolyte analyser for the direct measurement of urine concentrations of sodium, potassium, urea and creatinine

**DOI:** 10.1155/S1463924685000311

**Published:** 1985

**Authors:** D. A. Mallinson

**Affiliations:** Department of Chemical Pathology St. James's University Hospital Leeds LS9 7TF UK


Journal of Automatic Chemistry/*Journal of Clinical Laboratory Automation*, Vol. 7, No. 3 (Jul–Sep 1985), pp. 149–151

### A modification of the Hilger Analytical Chemispek multichannel electrolyte analyser for the direct measurement of urine concentrations of sodium, potassium, urea and creatinine

**D. A. Mallinson**

*Department of Chemical Pathology, St. James's University Hospital, Leeds LS9 7TF, UK*

### Introduction

The former procedure for urine analysis in this laboratory was to analyse both the neat sample and a 10-fold dilution using the Chemispek serum electrolyte analyser described by Smith *et al.* [1], and from these results to calculate the analyte concentrations. These values were then transcribed onto a report form. A greatly increasing work-load, largely from the Regional Renal Dialysis and Transplantation Unit at St. James's University Hospital, led to the development of a more rapid method of urine analysis with direct print-out of results.

### Methods

*Modification of Chemispek*

The IL543 flame photometer used on the Chemispek for sodium and potassium estimation has a range-expansion button which allows a 10-fold decrease in sensitivity. By using this facility for potassium, sodium and potassium were measured simultaneously. To eliminate the need for pre-dilution of urine samples a dilution circuit was introduced for the urea and creatinine channels (figure 1). The modification involves only the use of one sample

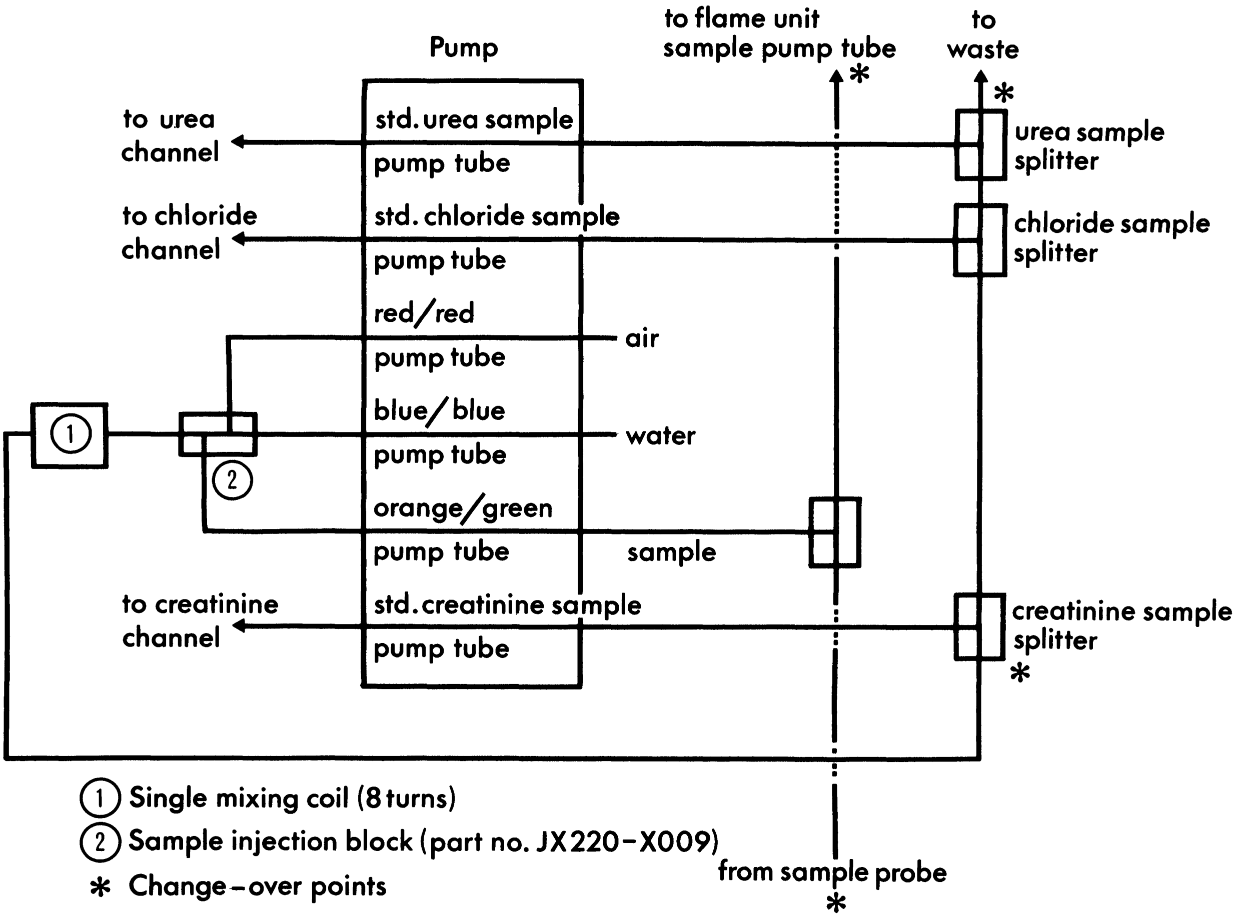

*Figure 1. Flow diagram for dilution circuit for urine electrolytes (for description see text).*

injection block, the connection of a transmission line from the sample probe to the sample splitter for the dilution circuit and of a line from the other side of the splitter to the flame unit sample pump tube. The bicarbonate channel sample splitter is disconnected and then the line from the dilution circuit connected to the creatinine sample splitter. The last alteration is to connect a transmission line from the urea sample splitter to waste. All that remains is to transfer standardization and other data into the microprocessor from the cassette unit. The whole procedure is rapid, taking well under 5 min both to convert the analyser for urine analysis and to convert it back for serum analysis.

The standards used for urine analysis are shown in table 1.

*Table 1. Concentrations of working standards.*

| Standard | Sodium (mmol/l) | Potassium (mmol/l) | Urea (mmol/l) | Creatinine (mmol/l) |
|---|---|---|---|---|
| 1 | 40 | 10 | 100 | 3·0 |
| 2 | 80 | 20 | 200 | 5·0 |
| 3 | 100 | 30 | 300 | 7·0 |
| 4 | 120 | 40 | 400 | 10·0 |
| 5 | 160 | 50 | 500 | 15·0 |

The updated microprocessor software allows the calculation of carry-over by the method of Broughton *et al.* [2], and also the use of a flexible plate format. The original plate format [1] was amended to utilize an aqueous drift standard (standard 3) at positions 1, 2, 21, 22, 41, 42, 59, 60 thus allowing more patient samples to be analysed per plate. The updated software also allows the use of a flexible label format for result printing. The microprocessor was programmed to print all urine results and normal ranges on to blank adhesive labels.

### *Carry-over*

This was assessed independently of the microprocessor using the method of Broughton *et al.* [2]. Aqueous standards 1 and 5 were used to represent low and high values respectively. Two plates were run using the following format—HHH LLL HHH LLL HHH LLL, where H = standard 5, L = standard 1.

### *Within-batch imprecision*

This was assessed using the protocol of McLelland *et al.* [3]. Three different aqueous solutions were used (standards 1, 3 and 5), representing low, medium and high levels respectively. Two plates were loaded according to the following format—DD HLH MMH LMH LLM HML MML DD HML LMH MLH HLH DD, where D = drift standard, L = low level, M = medium level, H = high level. This procedure was performed with and without carry-over correction.

### *Between-day imprecision*

This was assessed with the data from the daily analysis of a commercial freeze-dried urine (Fermtrol [4]).

### Results and discussion

The results are shown in tables 2, 3 and 4. The modification had similar imprecision to the alternative methods. Comparison with the unmodified Chemispek and with the Astra 4 showed excellent correlation (table 5).

*Table 2. Carry-over.*

| Analyte | High to low | Low to high |
|---|---|---|
| Sodium | 0·3% | 0·1% |
| Potassium | 0·0% | 0·0% |
| Urea | 1·4% | 1·8% |
| Creatinine | 2·9% | 4·7% |

*Table 3. Comparison of within-batch imprecision with and without carry-over correction.*

| Analyte | *N* | Approximate concentration (mmol/l) | With correction CV (%) | Without correction CV (%) |
|---|---|---|---|---|
| Sodium | 60 | 160 | 0·41 | 0·75 |
| | 60 | 100 | 0·30 | 0·42 |
| | 60 | 40 | 0·00 | 0·00 |
| Potassium | 60 | 50 | 1·01 | 1·03 |
| | 60 | 30 | 0·00 | 0·00 |
| | 60 | 10 | 0·00 | 0·00 |
| Urea | 60 | 500 | 1·52 | 1·62 |
| | 60 | 300 | 0·69 | 2·09 |
| | 60 | 100 | 1·55 | 3·50 |
| Creatinine | 60 | 15 | 1·86 | 3·02 |
| | 60 | 7 | 1·94 | 4·05 |
| | 60 | 3 | 0·88 | 5·30 |

*Table 4. Between-day imprecision values using commercial control.*

| Analyte | *N* | Mean concentration (mmol/l) | Range (mmol/l) | SD (mmol/l) | CV (%) |
|---|---|---|---|---|---|
| Sodium | 40 | 91 | 88–95 | 2·1 | 2·3 |
| Potassium | 40 | 36 | 33–37 | 0·7 | 1·9 |
| Urea | 40 | 117 | 104–123 | 3·9 | 3·4 |
| Creatinine | 40 | 7·9 | 7·5–8·2 | 0·3 | 3·1 |

The system has enabled the department to maintain daily analysis and reporting of results in the face of a 47% increase in work-load without any difficulties. The current work-load is about 140 samples a week. Analysis time has been halved, there is no initial manual dilution of samples, and the results are legibly printed thus eliminating transcription errors. The department has been able to halve costs, and the reagent costs are less than 10% of those for the Astra-4. The system has been in trouble-free operation for several months with no operator difficulties.

*Table 5. Correlation with alternative methods.*

| | | Modified Chemispek *versus unmodified Chemispek* | | | Modified Chemispek *versus Astra-4* | | |
|---|---|---|---|---|---|---|---|
| Analyte | *N* | *r* | slope | intercept | *r* | slope | intercept |
| Sodium | 25 | 0·99 | 1·01 | −0·10 | 0·99 | 0·98 | 0·90 |
| Potassium | 25 | 0·99 | 1·01 | −0·15 | 0·99 | 0·97 | 0·82 |
| Urea | 25 | 0·99 | 1·00 | 4·56 | 0·99 | 0·96 | 2·63 |
| Creatinine | 25 | 0·99 | 0·99 | 0·05 | 0·99 | 1·00 | −0·09 |

*r* = correlation coefficient.
Slope and intercept were calculated by method of Deming [5].

### References

1. Smith, M., Little, A. J. and Payne, R. B. *Annals of Clinical Biochemistry*. **18** (1981), 15.
2. Broughton, P. M. G., Gowenlock, A. H., McCormack, J. J. and Neil, D. W. *Annals of Clinical Biochemistry*, **11** (1974), 207.
3. McLelland, A. S., Fleck, A. and Burns, R. F. *Annals of Clinical Biochemistry*, **15** (1978), 12.
4. G. S. Ross Ltd, Unit 13, Fence Avenue, Industrial Estate, Macclesfield, Cheshire, UK.
5. Cornbleet, P. J. and Gochman, N. *Clinical Chemistry*, **25** (1979), 432.

**ROYAL SOCIETY OF CHEMISTRY ANALYTICAL DIVISION: THE INTEGRATED APPROACH TO LABORATORY AUTOMATION**

*A review of analytical instrumentation, robotics, laboratory information management systems and laboratory design and staffing: to be held at the Dormy Hotel, Ferndown, Dorset, UK, 23 to 25 October 1985*

This two-day residential seminar, organized by the Automatic Methods Group, will allow practising analysts, managers and directors, responsible for laboratory operations, to gain an in-depth appreciation of the impact of the computer revolution on the future of analytical chemistry. The seminar will commence by reviewing *analytical instrumentation*, examining current technology and likely developments in the future. Improved control of instruments and the ability to network instruments into systems has allowed substantial progress towards improving data quality, and highlighted sample preparation and presentation as one of the remaining major sources of variability; the role that *robotics* will play in addressing these deficiencies will be discussed. The intelligence of these systems can be exploited through 'standard' interfaces by larger computers responsible for *laboratory information management systems* communicating information via corporate networks. The combined impact of these developments on both the laboratory environment and staff will be significant if the integration of these technologies is to be carried out cost-effectively, and is likely to lead to a radical change in approach to *laboratory, design and staffing*.

In conjunction with this seminar there will be displays and demonstrations and an exhibition of the latest available hardware and software.

*Further information can be obtained from Dr Clive Jackson, Honorary Secretary, Automatic Methods Group RSC/AD, Health & Safety Executive, 403 Edgware Road, London NW2 6LN. Tel.: 01 450 8911.*

